# Learning constraints in protein evolution beyond known substitution frequencies

**DOI:** 10.1093/molbev/msag270

**Published:** 2026-11-04

**Authors:** Raphaël Bricout, Hugues Roest Crollius, Auguste Genovesio

**Affiliations:** École normale supérieure, Institut de Biologie de l’ENS (IBENS), CNRS, INSERM, Paris 75005, France; École normale supérieure, Institut de Biologie de l’ENS (IBENS), CNRS, INSERM, Paris 75005, France; École normale supérieure, Institut de Biologie de l’ENS (IBENS), CNRS, INSERM, Paris 75005, France

**Keywords:** evolution, amino-acid sequences, deep learning

## Abstract

During the evolution of protein sequences, amino acid substitutions follow broad patterns that are well established, such as those induced by the physico-chemical properties of residues or the position of sites along the sequence. While these deviations from random changes have been exploited for practical applications, such as to simulate protein evolution or to score protein alignments, they are too general to predict the precise evolution of individual protein sequences. To investigate whether protein sequences contain contextual information about their subsequent evolution beyond that captured by conventional substitution statistics and evolutionary conservation, we trained deep learning models based on an Evolutionary Scale Modeling (ESM2) encoder to predict the rate, the position, and the nature of the amino-acid substitutions in the evolution of ancestral sequences and compared their performance to simpler and explainable heuristics. Our results show that predicting the rate of amino acid changes is achieved equally well by deep learning and by using evolutionary information in outgroup sequences. This suggests that the protein sequence does not contain information that significantly improves the predictions for this task. On the opposite, we show that deep learning models predict the position of these substitutions more accurately than statistics-based baselines. Predictions of the nature of substitutions largely outperform baselines, with over 37% accuracy overall. We observe that rapidly evolving sequences are harder to predict, and substitutions preferentially occur between chemically similar amino acids. Interestingly, predictive information is predominantly contained within a local sequence context of 16 residues. These findings indicate that the nature of substitutions in protein evolution is partly governed by predictive rules that remain to be discovered through a more accurate modeling that could describe evolutionary trajectories based on sequence context alone.

## Introduction

Homogeneity of evolution has been investigated since the pioneering study of molecular evolution by Zuckerkandl and Pauling (1965) with the idea of a molecular clock. This idea of a constant rate of evolution of biological sequences has since been improved with different “relaxed molecular clocks” (Lepage et al. 2007). Indeed, rates of molecular evolution are now known to vary between species and genes (Echave et al. 2016), but also according to the position of sites in protein sequences. In particular, functional domains are more stable and evolve more slowly. Residues in structural domains also evolve more slowly than the other positions, leading to an excess of substitutions at the N- and C-termini due to the relationship between the sequence and the 3D structure of the resulting protein, as demonstrated in our previous study (Bricout et al. 2023). The nature of the substitutions itself is also known to be nonuniform, with some amino acid changes more likely to occur than others. Scoring matrices used by sequence alignment algorithms, such as the BLOSUM62 matrix (Henikoff and Henikoff 1992), attempt to describe these changes but are agnostic to the position in the sequence. Protein sequence evolution has also been described through increasingly sophisticated probabilistic and mechanistic models. Beyond empirical substitution matrices, phylogenetic models can account for variations in evolutionary rates among sites and lineages, site-specific amino-acid preferences, and the respective contributions of mutation and selection (Halpern and Bruno 1998; Rodrigue et al. 2010; Echave et al. 2016). Other approaches explicitly consider dependencies among sites and the effects of sequence context on amino-acid preferences. These models provide increasingly realistic descriptions of the evolutionary process and are widely used to estimate evolutionary parameters, reconstruct ancestral sequences, and evaluate the likelihood of alternative evolutionary histories. However, they generally describe probability distributions over possible evolutionary changes rather than asking to what extent the particular substitutions realized along a given lineage can be predicted from the ancestral sequence and its evolutionary context. Here, we address this complementary question: how much information about the realized position and nature of future amino-acid substitutions is contained in protein sequences, and can a flexible sequence model extract predictive information beyond that captured by simple, interpretable evolutionary statistics?

To address this question, we used a deep learning model trained on multiple alignments of extant protein sequences. The development of deep learning techniques has spectacularly progressed over the last 15 years. Both the large amounts of data provided by genome sequencing projects (Buermans and Den Dunnen 2014) and the exponential increase in computing power now make it possible to train deep neural networks on vast biological datasets. Historically, early neural networks for biological sequences, like DeepSEA or Basset (Zhou and Troyanskaya 2015; Kelley et al. 2016), used standard deep learning techniques, with simple one-hot encoding, convolutional layers, and multilayer perceptrons (MLPs). Current models rapidly gain closer relevance to biological sequences, by leveraging Natural Language Processing (NLP) (Eraslan et al. 2019; Routhier and Mozziconacci 2022). These techniques include the use of a learned encodings to give a relevant representation of the input data, for example using positional and token embedding, and self-supervised training based on masked autoencoding to learn the semantics of sequences, as popularized in BERT (Kenton and Toutanova 2019). NLP also employ attention layers (Vaswani 2017), known to efficiently handle both local and long-range interactions without loss of performance, which is a valuable property for biological sequences analysis. A well-known model leveraging these techniques for three-dimensional protein structure prediction is AlphaFold (Jumper et al. 2021; Abramson et al. 2024). Other, more generic models such as Evolutionary Scale Modeling (ESM) (Lin et al. 2023; Hayes et al. 2025) are not designed for a specific predictive task, but generate informative representations of coding sequences, commonly referred to as embeddings.

In this work, we hypothesized that beyond the substitution biases already captured by global substitution statistics and residue conservation, the sequences contain additional information that improves the prediction of amino-acid substitutions. This may however only affect the neutral component of evolution (Kimura 1991), whereas positive selection is governed by more complex, context-dependent, and partly external factors that are not easily predictable. To test this hypothesis in the absence of ground truth since the future of a protein sequence is unknown, we developed and trained a deep learning model to predict amino acid sequences of a target species, given orthologous sequences of closely related species. Instead of using deep learning solely for prediction, we used it as a tool to quantify the degree to which evolution of coding sequences can be predicted, an approach not considered in previous studies (Jacob 1977; Stern and Orgogozo 2009). Because our objective was not to estimate evolutionary parameters or substitution probabilities, but to quantify the predictability of realized substitutions, we compared the deep-learning models with interpretable predictors representing progressively richer sources of evolutionary information. Our findings show that while the deep neural network only performs only marginally better than simply relying on evolutionary conservation for the prediction of substitution positions, its accuracy is much higher when predicting the nature of the substitutions. It achieves more than 50% accuracy achieved for 60% of the most common amino acid substitutions. Our results also suggest that the learned rules do not depend on complex, long-range interactions.

## Results

### Using machine learning models to study protein sequence evolution

We developed two deep learning models to study protein sequence evolution (Fig. 1d). The first model, DeepEvoSeq, is based on transfer learning using a pretrained version of ESM2 for sequence embedding completed by additional trainable layers. The second model DeepEvoSeqSimple, is a simpler variant without ESM2 encoding (see the Methods section for more details). These two models, which differ in complexity, were both compared to deterministic baselines using simple and interpretable heuristics on three distinct prediction tasks: the substitution rate, the positions of substitutions, and the nature of the amino acid substitutions.

**Figure 1:**
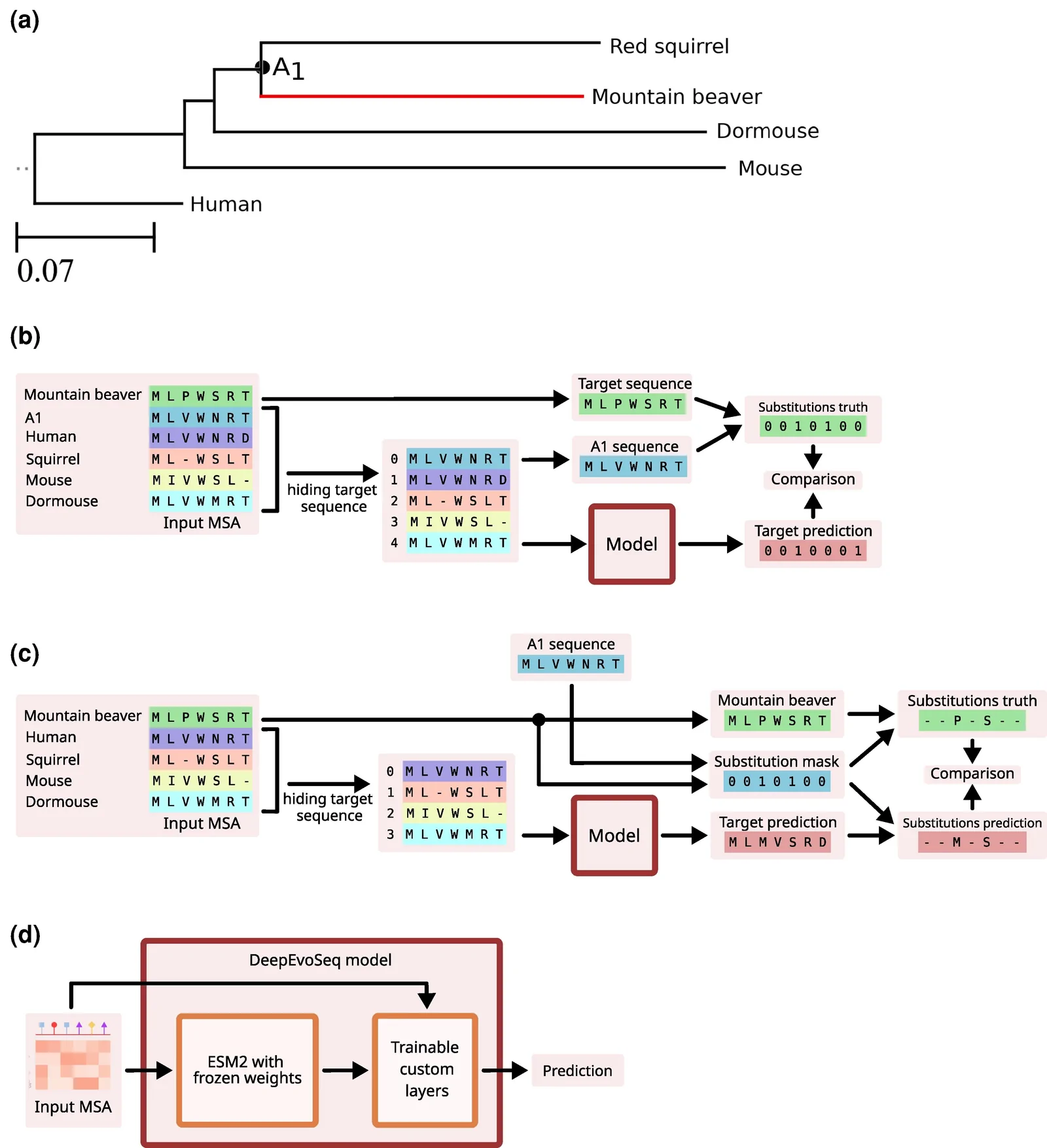
Rationale for the prediction tasks. a) Unrooted tree of species of interest. The branch where protein evolution is to be predicted is shown in red. A1 represents the reconstructed sequence of the common ancestor of the mountain beaver and its closest neighbor for coding sequences, the red squirrel. Scale is the rate of nucleotide substitutions per site. b) For position prediction, sequences of A1, the red squirrel and additional sequences from closely related species are used as input. The target sequence (mountain beaver) is not used in the model, but is used to compare the predictions with the ground truth. c) For amino acid substitution prediction, the A1 sequence is not used as data input. It is only used within the loss to define the positions of substitutions. d) Architecture of DeepEvoSeq: a frozen ESM2 embedding, and additional trainable attention and linear layers. DeepEvoSeqSimple consists only of the trainable custom layers.

The models were trained to predict protein sequences of the mountain beaver, because proteins from this species were not part of the ESM2 training that we use as an encoder, yet its genome and proteome were made publicly available at the beginning of the present study. To achieve this, we reconstructed the sequences of the common ancestor, called A1, of the mountain beaver and the red squirrel, the closest species for which a predicted proteome was available. The species tree calculated using genome assemblies from the NCBI, using CASTER (Zhang et al. 2025), is shown in Fig. 1a. The objective was then to predict the evolution of protein sequences that took place along the A1-beaver branch, shown in red in Fig. 1a, with the aim of predicting sequences more similar to beaver proteins than to red squirrel proteins.

Instead of predicting the evolution of each complete sequence, the prediction was split into three simpler tasks. The first task was to estimate the substitution rate for each sequence. The second task consisted of predicting the positions of substitutions on the A1-beaver branch, using the A1 sequence and an alignment of orthologous sequences from closely related species (Fig. 1b). Finally, the third task was to predict the nature of the substitutions on the A1-beaver branch, given their positions and an alignment of closely related species (Fig. 1c).

We performed these tasks on a dataset of 12,803 reference-based protein alignments of orthologous sequences. The mountain beaver sequence (our target) was used as a reference to align three closely related species, as well as the human sequence as outgroup. The A1 sequence was added to these sequences depending on the task. This protein sequence dataset was split into training and validation sets at an 80/20 ratio using MMseqs2 clusters (Steinegger and Söding 2017). The performance of these models on each of the three tasks led us to the following results on protein evolution.

### Sequences do not improve prediction of substitution rate

The substitution rate between sequences of A1 (the closest ancestor in our tree) and of the mountain beaver can be estimated using the red squirrel as a reference. A simple heuristic consists of, for each sequence, multiplying the substitution rate between the squirrel and the human sequences by the constant ratio $d_{SB}/d_{SH}$, where $d_{SB}$ is the branch length between the squirrel and the beaver species, and $d_{SH}$ is the branch length between the squirrel and the human species (Fig. 1a). Assuming similar branch lengths for the A1-beaver and the A1-squirrel branches, this value is then halved to obtain an estimate of the A1-beaver substitution rate. Attempts to enhance this simple prediction with our deep learning model did not result in a significant improvement over this baseline. Using the mean squared error as the loss function, the neural network models resulted in an error similar to that obtained from the heuristic method based on $d_{SB}/d_{SH}$. No substantial variation was observed when different models and loss functions were employed (see Table S1).

### The predictability of substitution positions is limited.

To predict substitution positions, we compared our deep learning models (Fig. 1d) with four baselines: “random,” “proxy,” “mouse,” and “consensus.” “Random” predicts a substitution with the same chance for all positions, resulting in a uniform distribution of substitutions across all positions. “Proxy” is based on the alignment using all species except A1 and the mountain beaver. It predicts substitutions at the most variable positions in this alignment. “Mouse” copies the sequence of the mouse, predicting substitutions at the positions where the mouse and A1 sequences differ. Finally, “consensus” builds the consensus sequence from the alignment excluding the A1 and mountain beaver sequences, and predicts substitutions at positions where this consensus sequence and the A1 sequence differ. A more detailed description of these baselines can be found in the Methods section.

Because the average amino-acid substitution rate between the A1 ancestor and the mountain beaver sequences is 4.9%, a marked imbalance exists between the frequency of positives (positions that underwent a substitution) and negatives (positions that did not). This imbalance makes simple and intuitive metrics, such as accuracy, irrelevant. Instead, more informative yet standard binary classification indicators were computed, including the precision-recall curve (Fig. 2a) and the ROC curve (Fig. 2b).

**Figure 2:**
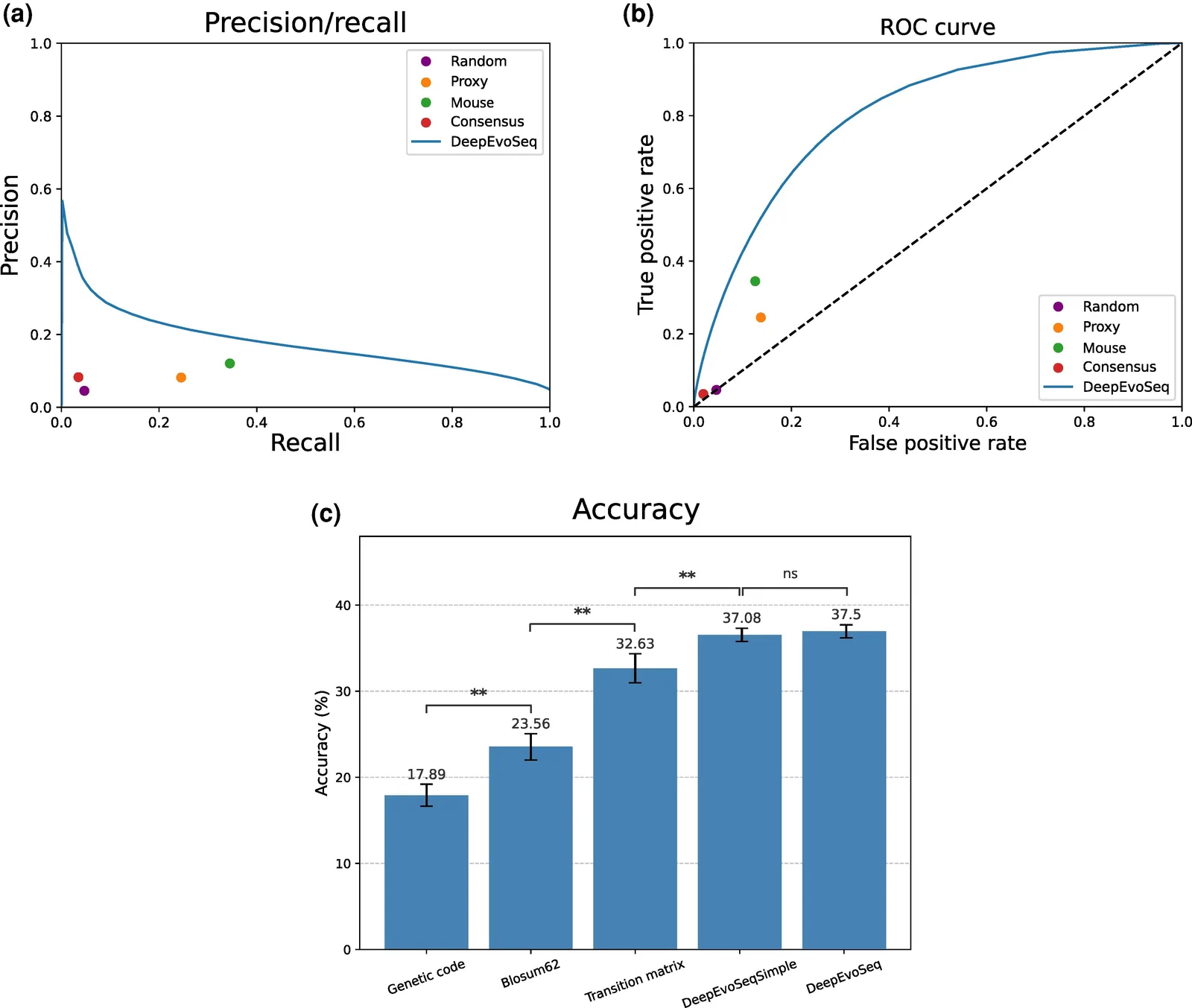
Performance of DeepEvoSeq compared to the baselines. For the prediction of the positions of substitutions, a) and b) are the precision/recall and the ROC curve, respectively. c) Mean accuracy for the prediction of the nature of amino acids given the positions of substitutions, averaged overall sequences.

The DeepEvoSeq model demonstrated an improvement in predicting the position of substitutions over the best baseline (the mouse sequences). Indicators such as F-score, the phi coefficient (Matthews 1975), or Youden's J statistic (Youden 1950) produced similar results as the ROC and precision-recall curves. Not using A1 in the input data slightly reduces the model's performance (see Table S2). We also confirmed that the distribution of the predicted substitution positions along the protein sequences matched the true distribution (see Figure S4).

### Amino acid substitutions can partly be predicted from the sequence

In order to focus on the predictability of the nature of amino acid changes only, we provided the network with the true positions of substitutions. The accuracy of the model was then compared to three baselines we named “Genetic code,” “BLOSUM62,” and “Transition matrix.” “Genetic code” predicted the amino acid substitutions as the most frequent amino acid resulting from a single nucleotide change according to the genetic code. “BLOSUM62” predicted the substitutions according to the maximal scores in the BLOSUM62 matrix. “Transition matrix” first learned transition probabilities in the A1-beaver branch, and used these to predict the substitutions. Accuracy was defined by calculating the mean accuracy over all positions with substitutions of each sequence, with equal weight assigned to each sequence regardless of the number of substitutions (Fig. 2d). In this case, the error bars represent 95% confidence intervals computed from the distributions of accuracies.

If substitutions were random, each amino acid could switch to any of the 19 other possible ones with a probability of 1/19=5.3%. The accuracy of this baseline is significantly lower than that of the three naive baselines. As expected, “Transition matrix” is the most accurate baseline and shows the highest predictive power, with over 32.6% accuracy. Context-specific variants of the “Transition matrix” reference model, using 3- or 5-mer sequences were tested, but performed worse than the default “Transition matrix” model, reaching accuracy of 32.9% for the 3-mer model and 30.1% for the 5-mer model. DeepEvoSeq outperformed all baselines, reaching 37.5% accuracy, meaning that more than a third of the substitutions were accurately predicted. A small improvement was observed when using the ESM2 encoder (DeepEvoSeq) compared to the simpler model without encoder (DeepEvoSeqSimple). No significant difference was observed when using attention layers or only linear layers for the additional trainable layers. This indicates that attention layers do not enhance the model's predictive performance and suggests that long-distance interactions play only a marginal role in the prediction of the nature of substitutions. Details about these experiments are in the supplemental material. Finally, we tested more standard models by turning probabilities into predictions. LG (Le and Gascuel 2008), a standard empirical amino acid replacement matrix, and a site-heterogeneous mutation–selection model implemented in PhyloBayes-MPI (Rodrigue et al. 2010), were tested but showed accuracies lower than the “Transition matrix” model.

### Robustness across specific and models

To ensure that the results are not specific to the mountain beaver branch, we conducted the same experiments using a different species. The choice to use a species that was not present in the databases used to train ESM2 severely limited the possible target species for this task, as well as the generality of our conclusions. We selected *Microgale talazaci* (Talazac's shrew tenrec), an endemic species from Madagascar, as the target species. The results, detailed in Figure S7, are very similar for both the position and the nature of the substitutions.

Finally, to ensure that the chosen model does not introduce biases, we performed similar experiments using the AlphaFold2 model (Jumper et al. 2021) without structure modules as the encoder for DeepEvoSeq instead of the ESM2 model. The results, detailed in Figure S6, show no significant differences for all the tasks.

We next conducted a series of experiments to characterize how the deep learning model improves prediction accuracy relative to the baselines. The performance of the model was evaluated by comparing it to the “Transition matrix” baseline, which was identified as the most accurate one.

### Rapidly evolving positions are harder to predict

We first examined whether the true evolutionary rate of protein sequences correlates with the accuracy of our model's predictions of amino acid substitutions. Indeed, it is expected that faster evolving proteins may be subject to fewer sequence constraints, and therefore have less predictable substitutions. The results (Fig. 3a) show that substitutions in sequences with low substitution rates are significantly better predicted. The accuracy values for the sequences with the top 50% and bottom 50% of sequences by substitution rates are shown in Table 1.

**Figure 3:**
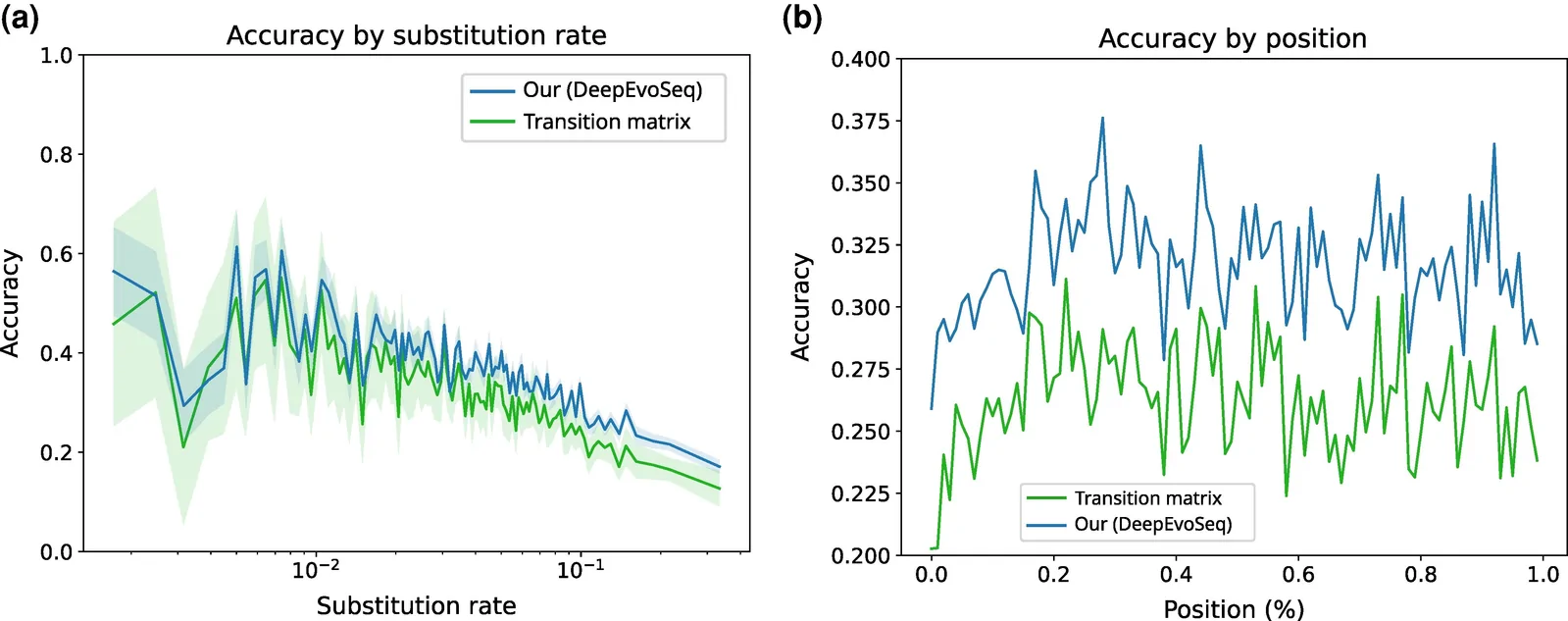
Characterization of accuracy comparing DeepEvoSeq with the best baseline “Transition matrix”, according to a) substitution rate (with 95% confidence intervals) and b) position along the sequence. Accuracy is not averaged by sequence but by position, lowering the accuracy values. The x axis shows the position of the substitutions from N- (0%) to C-termini (100%) as a % of the complete length of the sequence.

**Table 1:** Accuracy of the nature of substitutions comparing the DeepEvoSeq to the substitution matrix baseline, specifically for the 50% sequences with the highest and lowest substitution rates.

	All sequences	50% sequences with highest substitution rate	50% sequences with lowest substitution rate
Transition matrix baseline	32.6%	26.5%	38.8%
DeepEvoSeq	37.5%	32.1%	42.9%

To test the hypothesis that prediction accuracy is dependent on sequence constraints, prediction accuracy was plotted as a function of position within the sequence, which is known to correlate with structure-related constraints (Bricout et al. 2023). The results presented in Fig. 3b indicate that substitutions are more accurately predicted at central positions within the sequences.

### Rare substitutions are harder to predict

Amino acids have different chemical properties, and substitutions may not be predicted equally well due to their variable effects on protein structure and function (Xia and Li, 1998). To explore this, a confusion matrix showing the accuracy of each possible amino acid substitution was computed for the model and for the “Transition matrix” baseline (Fig. 4a-b). In this matrix, the frequency of correct predictions are shown in the diagonal cells (true positives) while incorrect predictions are shown outside of the diagonal (false positives). All values are normalized by row.

**Figure 4:**
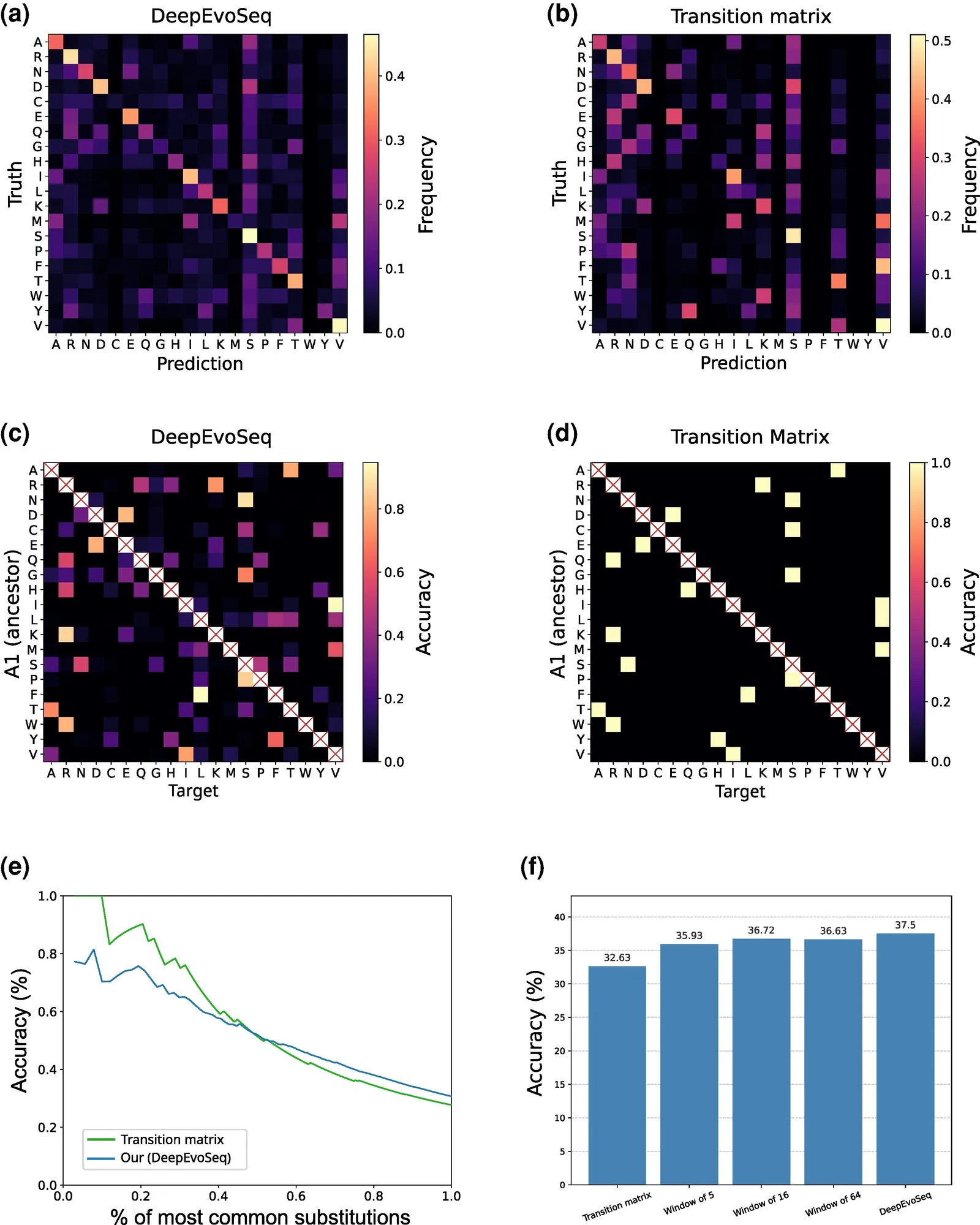
Analysis of the predictions of the nature of the substitutions. a) and b) show the confusion matrix for DeepEvoSeq and the “Transition matrix” predictions. The rows represent the true amino acids of the beaver sequences while the columns represent the predicted amino acids, and the value is the accuracy. Values on the diagonal are correctly predicted substitutions, and those outside are incorrect predictions. To compensate for the imbalance of amino acids in the data, the confusion matrix was normalized per row. c) and d) show the accuracy of the predictions for all possible amino acid substitutions between A1 and the beaver. The “Transition matrix” baseline is used with only the highest frequencies to maximize the accuracy, resulting in a binary matrix. e) shows the accuracy for DeepEvoSeq and the “Transition matrix” strategy when considering the most common substitutions. f) shows the improvements of the results according to the number of amino acids visible by the network.

The visible diagonal shows that a significant proportion of substitutions are correctly predicted. However, an imbalance in substitution prediction is evident. Some amino acids such as serine (S) and threonine (T) are over-predicted, as indicated by the lighter columns, while others, such as tryptophan (W) or cysteine (C), are under-predicted, as indicated by the darker columns. The prevalence of serine (S) and threonine (T), which are among the most common amino acids, contrasts with the rarity of tryptophan (W) and cysteine (C), which possess more specific chemical properties and are thus less likely to be predicted as substitution outcomes by the neural network. Biases appear in the “Transition matrix” baseline, positively for A, R, N, S, and negatively for M, P, F, W, Y. This is due to the deterministic nature of this baseline induced by the use of argmax, which rather than predicting amino acid according to transition probabilities, selects only the most likely outcome. As a result, it favors common amino acids while effectively excluding rarer ones from being predicted.

To provide a more comprehensive overview of the results, the accuracy of substitutions between A1 and beaver was computed separately for each amino acid pair. The results (Fig. 4c-d) show that DeepEvoSeq is able to predict the full range of possible changes from any starting amino acid, while the transition matrix is, by design, fully deterministic. For example, DeepEvoSeq is 90% accurate on all Isoleucine (I) to Valine (V) substitutions, but only about 30% accuracy on Alanine (A) to Valine (V) substitutions. This point is confirmed in Fig. 4e: the accuracy is higher for deterministic strategies such as the “Transition matrix” baseline when considering only the few most common substitutions, but DeepEvoSeq performs better at generalizing for rarer substitutions. However, many rare substitutions, for instance those resulting in tryptophan (W) are not well predicted, as shown by the dark column in Fig. 4c, perhaps in part because of the reduced number of instances in the training dataset compared to other substitutions. In contrast, the most common substitutions, such as those predicted by the “Transition matrix” baseline, are predicted with high accuracy by the DeepEvoSeq model. Finally, to further characterize the difficulty of predicting the nature of substitutions, we examined the relationship between prediction accuracy and the chemical properties of amino acids. The results, detailed in Figure S2, indicate that substitutions between amino acids with highly different chemical properties have lower prediction accuracy compared to those between more similar amino acids. Notably, DeepEvoSeq predicts better a broader range of properties.

### Substitutions show weak dependency on long-range interactions

The DeepEvoSeq model, which incorporates a complex architecture, did not demonstrate significantly superior performance (Fig. 2c) with respect to long-range interactions compared to the DeepEvoSeqSimple model, which employs a simpler architecture (Fig. 1d). This finding suggests that the majority of the information driving the improved results is not attributable to complex, long-range interactions such as those that the DeepEvoSeq could possibly capture, but rather to local interactions. To explore this further, a series of DeepEvoSeq variants was implemented, wherein the sequences were clipped to fixed window sizes. To avoid bias toward sequence termini, substitutions were not necessarily located in the middle of the window. Figure 4f shows that the results gradually improve as the window size is increased, and that the majority of the improvement (from 32.6 for the “Transition matrix” baseline to 36.9 for the default DeepEvoSeq model) is reached using a window of 16, which is less than 5% of median protein sequence length (353 AA in eukaryotes from Nevers et al. 2023). This observation suggests that the predominant source of information used to predict amino acid substitutions is derived from local interactions.

## Discussion

We show that a substantial proportion of the evolution of a protein sequence could be predicted, with notable variations observed across the three defined tasks. The prediction of the substitution rate was not better than a simple estimation based on branch lengths. This suggests that compared to branch lengths, the amino acid sequences themselves do not contain much additional information that would improve the prediction of the substitution rate. For the prediction of positions, only minor improvements over baselines were observed in our model. This suggests that most of the information used to predict the substitution positions is largely captured by residue conservation and almost fully exploited by the baselines.

However, the results were particularly interesting with respect to the nature of amino acid changes. First, the “genetic code” baseline reached 17.9% accuracy, which is considerably higher than random chance (5.26%). This reflects the fact that nonsynonymous base mutations lead to nonrandom amino acid substitutions based on the structure of the genetic code. The improvement resulting from the “Transition matrix” baseline, calculated specifically for the squirrel-beaver branches, confirms that a significant proportion of evolutionary rules, even as simple as transition probabilities, are species-specific (Halpern and Bruno 1998; Echave et al. 2016). Finally, using deep neural network we showed that more than 35% of the substitutions types were predictable, largely outperforming all deterministic baselines. This suggests that the evolution of coding sequences follows evolutionary trajectories constrained by local amino acid sequences.

Our results should not be interpreted as implying that deep learning captures an evolutionary process fundamentally different from that represented by existing models of sequence evolution. Rather, the network may implicitly integrate several known sources of heterogeneity, including site-specific selective constraints, amino-acid preferences, mutation biases, structural constraints, and context-dependent effects of neighboring residues (Chi and Liberles 2016; Echave et al. 2016; Youssef et al. 2021). In this sense, our approach is complementary to mechanistic and probabilistic evolutionary models: instead of specifying the components of the evolutionary process a priori and estimating their parameters, we ask how much information about the particular substitutions realized along a lineage can be extracted directly from sequence context. The improvement over substitution-frequency and conservation-based predictors therefore provides evidence for contextual predictive information not captured by these simple baselines, but does not by itself identify the evolutionary mechanisms responsible for that information.

It should be noted that the specific framework used in this study has several limitations. First, we used ancestral sequence reconstruction, where multiple hits and reversions can at best be estimated, so any reconstruction method will necessarily be imperfect. As this phenomenon is much rarer than forward substitutions (McCandlish et al. 2016), it does not prevent us from drawing general conclusions.

A second point relates to the technique used, which relies on a model of evolution. One consequence of this ancestral reconstruction is the potential circularity in the data: reconstructing the ancestral A1 sequence requires knowledge of the mountain beaver sequence, introducing a potential risk of circular reasoning. However, when predicting the nature of the substitutions, A1 was only used in the loss function, not in the training data, so there is no circularity issue. When predicting the positions of the substitutions, the sequence of A1 was used as input to the neural network, but here the objective was to predict information about the beaver sequence that is precisely not contained in A1, thus circumventing circularity.

Some experiments showed that in addition to the position in the sequence and to the substitution rate (Fig. 3a-b), chemical properties of amino acids impact the accuracy of predictions (Figure S2). Together, these observations highlight that multiple factors contribute to prediction performance. Further experimentation using structure annotations such as solvent accessibility (Ramsey et al. 2011; Wei et al. 2017) would be necessary to better understand and connect these observations. However, we note that long-range sequence information beyond 16 residues from the mutation have a minimal impact on the predictive power of the model. This may suggest that epistatic interactions contained within the protein sequence, such as those expected from the 3D structure of the protein, have a small influence on sequence evolution.

Finally, no extensive hyperparameter search or neural network optimization was performed. This may be an area where improvements could be made. The absence of a significant difference between DeepEvoSeq and DeepEvoSeqSimple suggests that either there are few long-distance interactions, or that the information from the ESM2 encoder and attention layers may not have been fully exploited.

Altogether, we show that a significant fraction of protein-coding sequence evolution can be predicted based on coding sequences, but it does not imply that the underlying biological process is deterministic. A portion of the observed predictability rely on simple transition rules, as demonstrated by the strong performance of the “Transition matrix” baseline. Another portion appears to follow more complex, variable patterns that are effectively captured by the DeepEvoSeq model with the use of contextual data from multiple sequence alignments. DeepEvoSeq may exploit statistical regularities arising from structural constraints such as secondary structure or solvent accessibility, mutation biases, purifying selection or lineage-specific evolutionary pressures. Distinguishing among these mechanisms will require additional analyses beyond predictive performance. Additionally, more complex mechanisms might exist, but due to the limitations of our modeling framework, their contribution may still be underestimated. A large part of the remaining substitutions, which represent more than 60% of the accuracy, might either reflect more stochastic process, with multiple equally likely substitution probabilities, or positive selection. This raises the question of differentiating between rules that govern substitutions with and without functional impact. Indeed, it would be interesting to focus specifically on substitutions that are relevant for phenotypic evolution, as done in (Stern and Orgogozo 2009), to determine whether such sites are more or less predictable than others.

## Methods

### Construction of the dataset

The choice of species was critical because sequences of the target species must be excluded from the input data to circumvent circularity during the model's training. Using a model based on ESM2, which has been pretrained on extensive public databases, precluded the use of any species included in these databases as the target species. Consequently, the mountain beaver (*Aplodontia rufa*) was selected as the target species for prediction. Its genome assembly (GCA_004027875.1) had been used for gene structure and proteome prediction available through the TOGA database (Kirilenko et al. 2023) (https://genome.senckenberg.de/download/TOGA/human_hg38_reference/Rodentia/Aplodontia_rufa__mountain_beaver__HLaplRuf1/). A dataset was constructed, comprising 12,803 alignments of protein sequences, including those from the mountain beaver (*A. rufa*), red squirrel (*Sciurus vulgaris*), mouse (*Mus musculus*), dormouse (*Muscardinus avellanarius*), and human (*Homo sapiens*). Each alignment contains all of these species, resulting in a number of alignments smaller than the number of proteins annotated in each genome individually. The mountain beaver sequences were obtained from the Zoonomia project (*Nature 2020*), while the human, squirrel, dormouse, and mouse sequences were downloaded from UniProtKB (TrEMBL) (Suzek et al. 2015).

The alignments were obtained by pairwise alignments using BLAST and reciprocal best hits on these four species using the mountain beaver genome as the reference. The sequences of the beaver-squirrel closest ancestor (named A1; see ancestral reconstruction) were incorporated into the alignments in the dataset. The dataset was then clustered using MMseqs2 (Steinegger and Söding 2017), and divided into training and validation subsets with an 80/20 ratio according to the MMseqs2 clusters to avoid similarity leakage. During training, the protein structures and the alignments (excluding the beaver sequence) were given to the network, and the prediction was compared to the true beaver sequence.

### Ancestral reconstruction

To obtain pairs of past/present sequences, A1 sequences were reconstructed. Ancestral sequences were obtained using the mountain beaver and the red squirrel, together with other closely related species—common dormouse (*M. avellanarius*), mouse (*M. musculus*), and human (*H. sapiens*)—used as outgroups. We employed BppAncestor (Guéguen et al. 2013), a method based on maximum likelihood estimation (MLE). We used LG08 (Le and Gascuel 2008) as the substitution model and compared it with JC69, LG08_F, and WAG01_F. These models produced similar reconstructions, with the exception of JC69, which is known to be simpler. Comparison details are shown in Figure S5.

### Neural network architecture

Our deep learning model, called DeepEvoSeq, consists of two main parts as shown in Fig. 1d. The first part is a fixed transformer encoder based on the ESM2 architecture (Jumper et al. 2021). It takes as input an alignment from the dataset of size 12,803 defined previously. Alignments are truncated or padded to obtain a fixed length *n_seq_*. The second part consists of additional trainable layers: two attention layers or linear layers, and final linear layer. We tested several versions with some of these layers removed, with no significant impact on the results. To limit computational cost, since attention layers scale quadratically with sequence length, we restricted the sequence length to $n_{seq}=1024$, randomly clipping longer sequences and padding shorter ones.

A simpler version of this model without the ESM2 encoder, called DeepEvoSeqSimple, was used as an ablation study to assess the relative importance of the encoder part.

There were some implementation differences for the different prediction tasks. For the positions of the substitutions, the output is an array of size $n_{seq}\times 2$, each position containing two values: one for substitution and one for nonsubstitution. We used a weighted cross-entropy loss (*w* = 10) on this output to account for the imbalance between positive and negative substitutions and to avoid mode collapse (i.e. predicting only negative substitutions). For inference, we computed the argmax for each position to define predictions of positive and negative substitutions. The number of substitutions predicted by the model is unconstrained, i.e. the model uses the input data to make these predictions. We also tried to add an additional loss term for substitution rate to improve the overall predictions, but it did not significantly improve the results. The effect of this loss is described in Figure S3.

For the nature of the substitutions, the output was an array of size $n_{seq}\times 21$, where each position represented a probability distribution over the 20 amino acids plus a gap. The cross-entropy loss was unweighted due to the approximate balance in the classes. For inference, we computed the *argmax* for each position to predict the amino acids.

The programming language was python, with PyTorch as the main library for the deep learning part.

### Baselines for the prediction of positions

We employed simple baselines that did not depend on trainable models, but rather on simple deterministic heuristics, in order to assess the added accuracy of using deep learning models. The baselines were adapted to each subtask. For the substitution positions, three baselines were defined. The first, most naïve baseline, called “random,” predicted substitutions uniformly in each sequence, matching the substitution rate. A slightly less naive baseline, called “mouse,” copied the substituted positions observed between the squirrel and the mouse, which are two closely related species. This baseline had the advantage of predicting substitutions using real biological data, but did not necessarily match the substitution rate in the beaver branch. The intuition behind this strategy is that positions undergoing substitution in a closely related branch are more likely to do so in the mountain beaver branch. We generalized this intuition by using a multiple sequence alignment rather than a single sequence and selecting positions with the highest substitution frequency, under the assumption that these positions were not well conserved and therefore generally more likely to undergo substitutions also in the beaver branch. This defined a third baseline, called “proxy.” A final baseline called “consensus” builds the consensus sequence defined by the alignment of all sequences, except A1 and the beaver. Predictions are then defined as positions differing between the consensus sequence and the A1 sequence. All the baselines were defined in a strictly binary manner, thus are not probabilities. In contrast, the deep learning models produced continuous outputs, allowing positive and negative predictions to be made in a continuous manner. For this reason, the baselines give only one value whereas the deep learning modals yield full ROC and precision-recall curves.

### Baselines for the prediction of amino acid changes

We also defined three baselines for the nature of the amino acid substitutions. In the first baseline “genetic code,” we considered single nucleotide substitutions in codons, where all four nucleotides are equally likely and all alternative codons are considered equally probable. This strategy used the genetic code to calculate the probability of each amino acid being substituted for another, based on the proximity of their respective codons. The most likely substitutions were chosen, with random selection used in cases of equal probability. The second baseline, called “BLOSUM62,” used the BLOSUM62 matrix (Henikoff and Henikoff 1992) as a transition matrix. The BLOSUM62 matrix was initially designed based on frequencies of different amino acid substitutions in highly conserved alignments of proteins from the BLOCKS database (Pietrokovski et al. 1996). It is thus a very general matrix based on alignments from a variety of species and specialized for conserved regions, i.e. regions more likely to be functional.

To maximize the prediction accuracy, the most likely substitution (the *argmax*) was used instead of the probabilities, with a random choice in cases of ties. For the third baseline, called “Transition matrix,” a substitution matrix based on the true substitution frequencies between the closest species (the squirrel) and the mountain beaver was computed from the training set. Unlike the BLOSUM62 matrix, which is very general, this matrix is specific to the species of interest and is therefore expected to yield better results.

## Supplementary Material

msag270_Supplementary_Data

## Data Availability

The data underlying this article are available in Zenodo at https://zenodo.org/records/23495397.

## Code availability

The code of DeepEvoSeq can be found at https://github.com/rabricout/DeepEvoSeq.
