# Supplementary material for: Learning constraints in protein evolution beyond known substitution frequencies
Source: Mol Biol Evol. 2026 Nov 4;43(11):msag270. doi: 10.1093/molbev/msag270 (PMC13756077; doi:10.1093/molbev/msag270)
Supplement: Supplementary file 1 [file msag270_supplementary_data.docx]

# Supplementary material

**Learning Constraints in Protein Evolution beyond Known Substitution Frequencies**

**Statistics for substitution rate prediction**

To predict the sequence-specific substitution rate, we compared the baseline with three variations of the DeepEvoSeq model: using L1 (mean absolute distance), L2 (mean squared distance), or Huber as the loss function. As metrics, we computed the L1 norm and L2 norm which should be minimised, as well as the R-squared (coefficient of determination), which should be maximised. The results are presented in Table supp. 1. The baseline slightly outperforms DeepEvoSeq across all three metrics.

|  | L1 | L2 (MSE) | R² |
| --- | --- | --- | --- |
| Baseline | $1.94\times{10}^{-2}$ | $1.51\times{10}^{-3}$ | $0.491$ |
| DeepEvoSeq (L1 loss) | $2.13\times{10}^{-2}$ | $1.72\times{10}^{-3}$ | $0.420$ |
| DeepEvoSeq (L2 loss) | $2.03\times{10}^{-2}$ | $1.60\times{10}^{-3}$ | $0.453$ |
| DeepEvoSeq (Huber loss) | $2.21\times{10}^{-2}$ | $1.67\times{10}^{-3}$ | $0.448$ |

Table supp. 1: metrics of the prediction of the substitution rate, comparing the variations of DeepEvoSeq to the baseline.

**Statistics for imbalanced data**
As a complement to the ROC curve and the precision-recall curve, we computed three other values: the F-score, the phi coefficient and Youden’s J. These indicators give values between 0 and 1 for the F-score and between -1 and 1 for the other two, and are more informative than a simple accuracy. The F-score is more common, but frequently criticized for not taking into account true negatives [1], thus the phi coefficient [2] or Youden's J [3] are typically preferred. The F-score is defined as follows:

$$F = \frac{2\times TP}{2\times TP+FP+FN}$$

The phi coefficient is known in machine learning as the Matthews Correlation Coefficient (MCC) and is interpreted in the same way as the Pearson correlation coefficient:

$$MCC = \frac{TP\times TN-FP\times FN}{\sqrt{(TP+FP)(TP+FN)(TN+FP)(TN+FN)}}$$

And Youden's J statistic is defined as:

$$J = \frac{TP}{TP+FN}+\frac{TN}{TN+FP}-1$$

The results are summarized in the Table supp. 2.

|  | F-score | Phi | J |
| --- | --- | --- | --- |
| Baseline random | 0.046 | 0.001 | 0.001 |
| Baseline consensus | 0.049 | 0.024 | 0.016 |
| Baseline mouse | 0.178 | 0.136 | 0.219 |
| Baseline proxy | 0.123 | 0.066 | 0.108 |
| DeepEvoSeqSimple (no A1) | 0.195 | 0.197 | **0.420** |
| DeepEvoSeqSimple | 0.234 | 0.221 | 0.397 |
| DeepEvoSeq (no A1) | 0.234 | 0.216 | 0.378 |
| **DeepEvoSeq** | **0.235** | **0.226** | 0.417 |

Table supp. 2: Values of F-score, phi and Youden's J indicators for baselines and DeepEvoSeq.

Performance is similar to that obtained with ROC and precision/recall curve, showing an improvement when using a deep learning model.

**ESM2 variations**

We tried several variations of the models to assess the importance of each part. We tried with no encoder (DeepEvoSeqSimple) and with several ESM2 encoders. “Small“ corresponds to esm2_t6_8M_UR50D (6 layers and 8M parameters), “medium” to esm2_t30_150M_UR50D (30 layers and 150M parameters), and “large” to esm2_t36_3B_UR50D (36 layers and 3B parameters).

|  | No encoder (DeepEvoSeqSimple) | ESM2 small | ESM2 medium | ESM2 large |
| --- | --- | --- | --- | --- |
| No additional attention | 37.1 | 37.4 | **37.5** | 36.8 |
| Additional attention | 37.0 | 37.3 | 37.3 | 35.3 |

Table supp. 3: Accuracy for nature of substitutions (%) for the different variations of the DeepEvoSeq.

**Global mean accuracy vs mean accuracy for each sequence**

For the nature of the substitutions, accuracy was calculated in two ways. The first method, used in the main paper (Figure 2.c, Figure supp. 1a), involved calculating the mean of the accuracies for each sequence, with equal weight attributed to each sequence regardless of the number of substitutions. In this case, the error bars represent 95% confidence intervals computed on the distributions of accuracies.

For the second method, the mean accuracy across all substitutions was calculated, with equal weight assigned to each substitution (Figure supp. 1b). The 95% confidence intervals were derived from three instances of the model. Given the deterministic nature of the transition matrix model, its error bar is 0.

Figure supp. 1: a) average of sequence accuracies. b) mean accuracy on all residues, regardless of the sequence

This result demonstrates that the average sequence accuracy is higher than the global average accuracy, suggesting that some sequences are more difficult to predict (see Figure 3b).

**Accuracy by amino acid properties**

To better interpret the values of the amino acid predictions, we grouped them according to amino properties as defined in Figure supp. 2d: for each pair of A1/beaver amino acid, we considered as an attribute substitution all the combinations of attributes between the two amino acids. For instance, a substitution from cysteine (C), which is small and aliphatic, to asparagine (N), which is small and polar, would be counted as 4 attribute replacements: small to small, small to polar, aliphatic to small, and aliphatic to polar. The result is an accuracy matrix presented for DeepEvoSeq in Figure supp. 2a, and for the “Transition matrix” baseline in Figure supp. 2b.

Figure supp. 2: Matrices of the accuracy based on amino acid attribute changes. a) for DeepEvoSeq. b) for the « transition matrix » baseline. c) difference between the two, highlighting the improvement (red values) of DeepEvoSeq compared to the baseline.

To highlight the improvements of DeepEvoSeq compared to the baselines, we have plotted in Figure supp. 2c the difference in accuracy of amino acid properties between DeepEvoSeq and the transition matrix baseline, for all pairs of A1/beaver amino acids. In this figure, the red values show the improvements of DeepEvoSeq compared to the baseline for most amino acids, while the blue values display better performance for the baseline. For example, transitions from aliphatic to tiny amino acids are predicted with about 20% more accuracy with DeepEvoSeq than with the transition matrix. The baseline has only one output for each amino acid in A1, so it was expected to perform better for some amino acid pairs. This figure shows that the neural network predicts better a broader range of properties (red outside the diagonal).

**Substitution rate Loss**

The substitution rate is highly variable from sequences to sequence. However when predicting the positions of substitutions, we noticed that our model predicted almost the same substitution rate for all sequences. To make our model more sequence-specific, we added a substitution rate loss $L_{SR}$, which penalize when the number of predicted substitutions does not match the substitution rate.

$$L_{SR}= \left\| \frac{1}{n_{seq}}\sum_{i \in[1, \ldots, n_{seq}]} {softmax}_{\varepsilon}\left( x_{i}, 1 \right)-s \right\|$$

With $x$ the predictions of the positions, $s$ the substitution rate, and ${softmax}_{\varepsilon}$ the generalization of the softmax defined as:

$${softmax}_{\varepsilon}\left( x, i \right)= \frac{exp({x_{i}}/\varepsilon)}{\sum_{j} exp({x_{j}}/\varepsilon)}$$

We used this function with $\varepsilon=0.1$ to sharpen the softmax while keeping a differentiable function so it can be used as a loss. We plotted in Figure supp. 2 the average ROC values and compared them to a line representing all the true substitution rate of the A1/beaver branch. Increasing the weight of the loss $w_{SR}$ brings the predictions closer to this line, but does not significantly improve the results.

Figure supp. 3: Average of the ROC values for a threshold of 0.5, for variations of DeepEvoSeq using different values of the weight of the substitution rate loss $\boldsymbol{w}_{\boldsymbol{SR}}$. The red line represents the true mean substitution rate (0.045).

**Amino Acid Baselines**

Amino acid baselines are defined in a deterministic way to maximise accuracy. In case of equally equivalent possibilities, an amino is selected randomly among them.

| Baseline => | Codons table | BLOSUM62 | Transition matrix |
| --- | --- | --- | --- |
| A | TGPSV | S | T |
| R | GS | K | K |
| N | K | DHS | S |
| D | E | E | E |
| C | S | A | S |
| Q | H | E | R |
| E | D | DQ | D |
| G | R | ANS | S |
| H | Q | Y | Q |
| I | L | V | V |
| L | FV | IM | P |
| K | N | R | R |
| M | I | L | L |
| F | L | Y | L |
| P | TLSAR | ADQEKST | S |
| S | TR | ANT | P |
| T | S | S | A |
| W | RC | Y | R |
| Y | FDSNHC | F | H |
| V | L | I | A |

Table supp. 4: Values of the most probable substitutions for the codon table, the BLOSUM62 matrix and for the A1/beaver transition matrix. Some values might be similar in the codons table or the BLOSUM62 matrix, leading to multiple equally likely substitutions for some amino acids.

**Distribution of substitution positions**

A side analysis consisted in verifying whether the predictions of substitution positions followed the correct distribution of substitutions, both in terms of substitution rates and positions along the sequences. The default threshold of 0.5 defining positives and negatives gave a high average substitution rate, so for the analyses of the results we also used a threshold of 0.75 which matches the true average substitution rate. Note that this threshold is specific to the model architecture and hyperparameters. The results presented in Figure supp. 4 show that the prediction follows this distribution, with almost identical values for a threshold of 0.75.

Figure supp. 4: Distribution of substitution rate by position for DeepEvoSeq with the default threshold of 0.5 and the threshold of 0.75, compared to the truth and to the mouse.

**Species Tree**

The species tree was constructed from alignments blocks using CASTER [4], giving the following Newick tree :

((((Sciurus vulgaris:0.169964,Aplodontia rufa:0.160192)100.0:0.021866,Muscardinus avellanarius:0.244278)100.0:0.013438,Mus musculus:0.268455):0.073197,Homo sapiens:0.073197) ;

**BppAncestor models**

To limit the bias coming from the ancestral reconstruction, we compared several substitution models used with BppAncestor.

Figure supp. 5: Comparison between different substitution models for the ancestral reconstruction. a) percentage difference of reconstructed amino acids between methods. b) fraction of sequences that are reconstructed exactly identical.

Figure supp. 5 shows that models perform similarly in reconstruction, with the exception of JC69, which is known to be a simpler, less realistic model. For this reason we chose LG08 as the substitution model to perform ancestral reconstruction with BppAncestor.

**AlphaFold2**

To assess that the design of the model was not a major flaw, we made a variation of DeepEvoSeq using a truncated version of AlphaFold2 [5] as the embedding part instead of ESM2 [6]. To this end, we kept the Evoformer blocks of Alphafold2 and removed the structure blocks, resulting in a MSA representation. We then added either trainable Evoformer blocks, either trainable simple linear layers to define the full model. Some additional modifications have been made due to the specificities of the architecture. We modified the maximum sequence length to 768, based the alignments on the human sequence and performed prior database searches for similar sequences.

Figure supp. 6: Same figure as figure 2, using a truncated part of Alphafold2 instead of ESM2 as the encoder. a) precision/recall curve for predictions of substitutions positions. b) ROC curve for predictions of substitutions positions. c) mean of accuracies for prediction of nature of substitutions.

Figure supp. 6 shows that the experiments made using Alphafold2 instead of ESM2 as an encoder did not significantly changed the results.

**Microgale**

To verify that the results are not specific to the target species (the mountain beaver), we performed similar experiments using a distinct set of species. We chose *Microgale talazaci*, a mammal from Madagascar, as the target species. This choice was made for the same reasons as for the mountain beaver: this species was not used to train ESM2 or AlphaFold2, but is available thanks to the Zoonomia project. We used *Chrysochloris asiatica*, *Elephantulus edwardii*, *Orycteropus afer*, *Procavia capensis*, and *Truchechus manatus* as other species for the alignment.

Figure supp. 7: Metrics for prediction of substitution positions and substitution natures, similarly to figure 2, but with *Microgale talazaci* as the target species instead of the mountain beaver. a) precision/recall curve for predictions of substitutions positions, with dots for baseline methods. b) ROC curve for predictions of substitutions positions. c) mean of the sequence accuracies for predictions of nature of substitutions.

Table supp. 4 and Figure supp. 7 show that results obtained using *Microgale talazaci* as the target species are very similar, yet even better, to those obtained using the mountain beaver, both for position and nature of substitution.

|  | F-score | Phi | J |
| --- | --- | --- | --- |
| Baseline random | 0.08 | 0.00 | 0.00 |
| Baseline consensus | 0.02 | 0.00 | 0.00 |
| Baseline closest species | 0.202 | 0.137 | 0.128 |
| Baseline proxy | 0.135 | 0.045 | 0.051 |
| **DeepEvoSeq** | **0.329** | **0.304** | **0.508** |

Table supp. 5: Values of F-score, phi and Youden's J indicators for baselines and DeepEvoSeq, with *Microgale talazaci* as the target species instead of the mountain beaver.

**Model architectures for substitution positions**

For all these architectures, variations have been tried using linear layers instead of attention layers. For DeepEvoSeq, proj_one_hot is the direct embedding of sequences while proj_embedding is the embedding of data embedded using ESM2. Hyperparameters are lr=0.001 and batch_size=32, optimizer is Adam.

**DeepEvoSeq**

```
 -> DeepEvoSeqPositionGeneric(
  (proj_one_hot): Linear(in_features=21, out_features=32, bias=True)
  (norm_raw): LayerNorm((32,), eps=1e-05, elementwise_affine=True, bias=True)
  (proj_embedding): Linear(in_features=640, out_features=32, bias=True)
  (norm_embedding): LayerNorm((32,), eps=1e-05, elementwise_affine=True, bias=True)
  (proj_in): Linear(in_features=256, out_features=64, bias=True)
  (dropout): Dropout(p=0.25, inplace=False)
  (blocks): ModuleList(
    (0-1): 2 x FeedForwardBlock(
      (norm): LayerNorm((64,), eps=1e-05, elementwise_affine=True, bias=True)
      (ffn): Sequential(
        (0): Linear(in_features=64, out_features=64, bias=True)
        (1): GELU(approximate='none')
        (2): Linear(in_features=64, out_features=64, bias=True)
        (3): GELU(approximate='none')
        (4): Linear(in_features=64, out_features=64, bias=True)
      )
      (drop): Dropout(p=0.25, inplace=False)
    )
  )
  (norm_out): LayerNorm((64,), eps=1e-05, elementwise_affine=True, bias=True)
  (classifier): Linear(in_features=64, out_features=2, bias=True)
)
```

**DeepEvoSeqSimple**

```
-> DeepEvoSeqPositionGeneric(
  (proj_one_hot): Linear(in_features=21, out_features=32, bias=True)
  (norm_raw): LayerNorm((32,), eps=1e-05, elementwise_affine=True, bias=True)
  (proj_in): Linear(in_features=128, out_features=64, bias=True)
  (dropout): Dropout(p=0.25, inplace=False)
  (blocks): ModuleList(
    (0-1): 2 x FeedForwardBlock(
      (norm): LayerNorm((64,), eps=1e-05, elementwise_affine=True, bias=True)
      (ffn): Sequential(
        (0): Linear(in_features=64, out_features=64, bias=True)
        (1): GELU(approximate='none')
        (2): Linear(in_features=64, out_features=64, bias=True)
        (3): GELU(approximate='none')
        (4): Linear(in_features=64, out_features=64, bias=True)
      )
      (drop): Dropout(p=0.25, inplace=False)
    )
  )
  (norm_out): LayerNorm((64,), eps=1e-05, elementwise_affine=True, bias=True)
  (classifier): Linear(in_features=64, out_features=2, bias=True)
)
```

**Model architectures for substitution natures**

**DeepEvoSeq**

```
 -> DeepEvoSeqGeneric(
  (proj_one_hot): Linear(in_features=21, out_features=32, bias=True)
  (norm_raw): LayerNorm((32,), eps=1e-05, elementwise_affine=True, bias=True)
  (proj_embedding): Linear(in_features=320, out_features=32, bias=True)
  (norm_embedding): LayerNorm((32,), eps=1e-05, elementwise_affine=True, bias=True)
  (proj_in): Linear(in_features=256, out_features=64, bias=True)
  (dropout): Dropout(p=0.25, inplace=False)
  (blocks): ModuleList(
    (0-1): 2 x FeedForwardBlock(
      (norm): LayerNorm((64,), eps=1e-05, elementwise_affine=True, bias=True)
      (ffn): Sequential(
        (0): Linear(in_features=64, out_features=64, bias=True)
        (1): GELU(approximate='none')
        (2): Linear(in_features=64, out_features=64, bias=True)
        (3): GELU(approximate='none')
        (4): Linear(in_features=64, out_features=64, bias=True)
      )
      (drop): Dropout(p=0.25, inplace=False)
    )
  )
  (norm_out): LayerNorm((64,), eps=1e-05, elementwise_affine=True, bias=True)
  (classifier): Linear(in_features=64, out_features=21, bias=True)
)
```

**DeepEvoSeqSimple**

```
 -> DeepEvoSeqGeneric(
  (proj_one_hot): Linear(in_features=21, out_features=32, bias=True)
  (norm_raw): LayerNorm((32,), eps=1e-05, elementwise_affine=True, bias=True)
  (proj_in): Linear(in_features=128, out_features=64, bias=True)
  (dropout): Dropout(p=0.25, inplace=False)
  (blocks): ModuleList(
    (0-1): 2 x FeedForwardBlock(
      (norm): LayerNorm((64,), eps=1e-05, elementwise_affine=True, bias=True)
      (ffn): Sequential(
        (0): Linear(in_features=64, out_features=64, bias=True)
        (1): GELU(approximate='none')
        (2): Linear(in_features=64, out_features=64, bias=True)
        (3): GELU(approximate='none')
        (4): Linear(in_features=64, out_features=64, bias=True)
      )
      (drop): Dropout(p=0.25, inplace=False)
    )
  )
  (norm_out): LayerNorm((64,), eps=1e-05, elementwise_affine=True, bias=True)
  (classifier): Linear(in_features=64, out_features=21, bias=True)
)
```

For all models, when using attention on trainable layers, instead of the FeedForwardBlock(), we use:

```
TransformerBlock(
  (norm_attn): LayerNorm((64,), eps=1e-05, elementwise_affine=True, bias=True)
  (attn): MultiheadAttention(
    (out_proj): NonDynamicallyQuantizableLinear(in_features=64, out_features=64, bias=True)
  )
  (drop_attn): Dropout(p=0.25, inplace=False)
  (norm_ffn): LayerNorm((64,), eps=1e-05, elementwise_affine=True, bias=True)
  (ffn): Sequential(
    (0): Linear(in_features=64, out_features=128, bias=True)
    (1): GELU(approximate='none')
    (2): Dropout(p=0.25, inplace=False)
    (3): Linear(in_features=128, out_features=64, bias=True)
  )
  (drop_ffn): Dropout(p=0.25, inplace=False)
)
```

**References**

[1]	D. Chicco and G. Jurman, ‘The advantages of the Matthews correlation coefficient (MCC) over F1 score and accuracy in binary classification evaluation’, *BMC genomics*, vol. 21, pp. 1–13, 2020.

[2]	B. W. Matthews, ‘Comparison of the predicted and observed secondary structure of T4 phage lysozyme’, *Biochimica et Biophysica Acta (BBA)-Protein Structure*, vol. 405, no. 2, pp. 442–451, 1975.

[3]	W. J. Youden, ‘Index for rating diagnostic tests’, *Cancer*, vol. 3, no. 1, pp. 32–35, 1950.

[4]	C. Zhang, R. Nielsen, and S. Mirarab, ‘CASTER: Direct species tree inference from whole-genome alignments’, *Science*, p. eadk9688, 2025.

[5]	J. Jumper *et al.*, ‘Highly accurate protein structure prediction with AlphaFold’, *nature*, vol. 596, no. 7873, pp. 583–589, 2021.

[6]	Z. Lin *et al.*, ‘Evolutionary-scale prediction of atomic-level protein structure with a language model’, *Science*, vol. 379, no. 6637, pp. 1123–1130, 2023.
